# Wnt-induced secreted proteins-1 play an important role in paraquat-induced pulmonary fibrosis

**DOI:** 10.1186/s40360-022-00560-y

**Published:** 2022-04-06

**Authors:** Lanrong Li, Shengnan Lv, Xin Li, Jingyan Liu

**Affiliations:** 1 Emergency Department, Linyi People’s Hospital, Linyi, China; 2 Outpatient Department, Linyi People’s Hospital, Linyi, China; 3 Emergency Department, Longgang District People’s Hospital of Shenzhen, No. 53 of Aixin Road, Longgang District, Shenzhen, 518115 Guangdong Province China

**Keywords:** Paraquat, Poisoning, Pulmonary fibrosis, WISP1, EMT, MMP-9

## Abstract

**Background:**

The objective of this article is to observe the expression of Wnt-induced secreted proteins-1 (WISP1) in paraquat (PQ)-induced pulmonary fibrosis (PF) to explore the role of WISP1.

**Methods:**

Healthy individuals were included in the control group. Patients who had acute lung injury or PF were included in the PF group. Venous blood samples were collected from the patients on days 1 and 3 following PQ poisoning to detect the expression levels of the WISP1 gene and protein concentration. Any changes in the patients’ blood gas analysis index were reviewed. In addition, chest computed tomography (CT) and x-ray images were observed to evaluate the relationship between WISP1 expression and disease severity.

**Results:**

The expression of the WISP1 gene and the serum WISP1 protein concentration were higher in patients with PQ poisoning combined with PF than in patients without PF (*P* < 0.01). Serum PQ concentration was positively correlated with WISP1 gene expression (*r* = 0.621, *P* < 0.01), and serum WISP1 protein concentration (*r* = 0.596, *P* < 0.01) was considered a risk factor [odds ratio (OR) = 4.356, *P* < 0.05] for PQ-induced PF. Concurrently, the results of the adjusted and non-adjusted OR value for WISP1 gene expression and WISP1 protein concentration on day 1 was, respectively, as follows: OR = 12.797, 95% confidence interval (CI) (2.478–66.076), *P* = 0.002, OR’ = 11.353, *P* = 0.005; and OR = 1.545, 95% CI (1.197–1.995), *P* = 0.001, OR’ = 1.487, *P* = 0.003. The CT scan of a 20-year-old male with PQ-induced PF (20 ml) was observed, and it showed a typical hyaline-like lesion in the lungs on day 22 after poisoning; on day 33 after poisoning, the lungs showed localised consolidation combined with air bronchography.

**Conclusion:**

The expression of WISP1 was higher in the patients with PQ-induced PF compared with the patients without PF. Accordingly, WISP1 plays an important role in PQ-induced PF.

## Background

Paraquat (1,1′-dimethyl-4,4′-bipyridinium dichloride; PQ) is a herbicide that is toxic to humans and animals [1]. PQ poisoning is common in developing countries. A person who ingests 20–40 mg/kg of PQ can experience multiple organ failure (liver, lungs, heart and kidneys) within several days, subsequently leading to death [2]. PQ poisoning often induces pulmonary fibrosis (PF); this, in turn, results in acute lung injury (ALI) and respiratory failure. Due to the lack of an effective treatment, the fatality rate of PQ poisoning ranges between 30.0–88.89% [3, 4].

PQ causes lung damage by promoting the apoptosis of lung cells, the degradation of the extracellular matrix (ECM), the up-regulation of the expression of inflammatory factors and the reduction in antioxidant gene expression. Recently, studies have shown that PQ can act on alveolar epithelial cells and induce their transformation into mesenchymal cells (epithelial–mesenchymal transition; EMT), giving rise to early PF [5–7]. EMT is characterised by a decrease in the expression of the epithelial marker E-cadherin and an increase in the expression of the mesenchymal marker vimentin. Additionally, the EMT process is related to the following cytokines: transforming growth factor (TGF)-β1, tumour necrosis factor-α (TNF-α) and interleukin-6 (IL-6) [9]. PQ can induce EMT production in the alveolar cells, and the TGF-β1 receptor antagonist can attenuate mesenchymal transformation and fibronectin secretion [8].

A newly discovered molecule in the Cyr61/CTGF/NOV (CCN) family of secretory signalling molecules, Wnt-induced secreted proteins-1 (WISP1), is critically involved in epithelial–mesenchymal crosstalk [9] and plays an important role in the interaction between epithelial and mesenchymal cells [10, 11]. WISP1 can induce fibroblast proliferation and ECM deposition in the pathological remodelling of asthma [12], and its concentration increases in patients with idiopathic PF and in lung fibrosis animal models [13, 14]. Furthermore, WISP1 expression is increased and induced by TGF-β1 in primary lung fibroblasts; however, TGF-β1-induced WISP1 messenger ribonucleic acid (mRNA) expression in lung fibroblasts is reversed by miR-30a and miR-92a [14]. TGF-β1 and TNF-α induces WISP1 mRNA, and protein secretion increases in a time- and concentration-dependent manner in primary human lung fibroblasts. Moreover, WISP1 is required for IL-6 expression by the profibrotic cytokines TGF-β1 and TNF-α [15]. EMT is implicated in early PQ-induced PF [5–7]. In addition, the WISP1 concentration increases in alveolar epithelial type-II cells with stretch-induced EMT in vitro [16] and is linked to ventilator-induced lung injury models in vivo [17].

No study has reported on the changes of WISP1 in patients with PQ-induced PF or the role of WISP1 in PQ-induced EMT. This study’s hypothesis is that WISP1 may influence PQ-induced EMT and may play a role in PQ-induced PF. Accordingly, the effect of WISP1 on PQ-induced EMT in vitro is observed. In addition, a prospective control study is conducted to observe the changes in WISP1 among PQ-poisoned patients and to analyse the correlation between WISP1 and PQ-induced PF.

## Methods

The current research was a case-control prospective study. Patients with PQ-poisoning, who were admitted to our hospital from January 2013 to June 2014, were selected. A total of 37 cases were included.

### Participants

Ten healthy patients were included in the control (C) group and were tested for serum PQ concentration and WISP1 gene and protein expression. Thirty-seven patients who ingested 20% (w/v) PQ (from January 2013 to June 2014) were included in the study. The project was approved by the Shandong Provincial Health and Family Planning Commission at the end of 2011, and the pre-experiment was performed in 2012. From 2013 to 2014, patients were enrolled in the experimental study. At this stage, the department received the highest number of PQ-poisoned patients. In 2014, the clinical research phase of the project was completed, and the in vitro experimental research followed. Therefore, only admitted patients, from 2013 to 2014, were selected. Subsequently, with the national ban on the production and use of PQ, PQ-poisoned patients decreased annually. Accordingly, there were no new patients in the follow-up group. The protocols of this study followed the Declaration of Helsinki and were approved by the Ethics Committee of Linyi People’s Hospital (approval no. 2013-J-03).

### Inclusion and exclusion criteria

Inclusion criteria: 1) Patients diagnosed with occupational acute PQ poisoning (GBZ 246–2013); 2) the degree of PQ ingestion was defined as one mouthful (up to 20 ml); 3) patients whose serum could detect PQ; 4) poisoning within 24 h of admission; and 5) patients who voluntarily signed informed consent forms.

Exclusion criteria: 1) Patients with a low intake of PQ; 2) patients who did not swallow after oral administration; 3) patients who took other drugs or had been poisoned in other ways; and 4) patients with additional underlying diseases.

### Groups

The serum PQ levels of all the participants were measured. The participants were randomly divided into the following three groups: (1) a healthy human group (negative control, group C, *n* = 10); (2) an ALI or PF group (PF group, *n* = 13); and (3) a non-PF complications group (NPF group, *n* = 24).

### Blood sample collection

Venous blood samples (6 mL) were collected from each patient on days 1 and 3 following PQ poisoning. The blood samples were centrifuged at 3000×*g* for 10 min, and the serum was collected and stored at − 80 °C until analysis.

### Reverse transcription-polymerase chain reaction assay

There were 13 patients in the PF group on day 1 of poisoning. Reverse transcription-polymerase chain reaction (RT-PCR) was performed for the 11 surviving patients in the PF group on day 3. Additionally, the 10 patients in group C were also measured in terms of WISP1 gene expression with RT-PCR. There were 24 patients in the NPF group on day 1 and RT-PCR was performed for all 24 patients on day 3.

In a 15 ml centrifuge tube, a 2 ml anticoagulant was added to a 10 ml erythrocyte lysate and incubated on ice for 20 min before vortex oscillation was conducted three times. The supernatant (at 4 °C) was removed by centrifugation (10 min, 2000 revolutions). Then, 4 ml red blood cell lysates were added to the peripheral blood in a transient suspension of neutrophil cells. The ribonucleic acids (RNA) of the neutrophil granulocytes were extracted using a TRIzol reagent (Sigma-Aldrich Co. LLC). Both spectrophotometry and agarose gel electrophoresis were performed to measure the total RNA concentration. The WISP1 gene was tested via RT-PCR assays [14]. The primers used in this study were purchased from Invitrogen Life Co., Shanghai, China (Table 1).

**Table 1:** The primers that were used for RT-PCR assays

Gene	Sequence	
GADPH	Forward	5′-GGAGCGAGATCCCTCCAAAAT-3′
	Reverse	5′-GGTACTCTTCATACTGTTGTCGG-3′
WISP1	Forward	5′-TGCTGTAAGATGTGCGCTCAG-3′
	Reverse	5′-GCTACCCCATGCGTTATCCTCACA-3′

### Enzyme-linked immunosorbent assay

The WISP1 protein levels in the blood serum of the patients were determined using a commercial enzyme-linked immunosorbent assay (ELISA) kit (Abcam, USA) according to the manufacturer’s instructions. ELISA was performed on the PF group (13 patients on day 1 and 11 patients on day 3). There were 24 patients in the NPF group on day 1 of poisoning, and ELISA was performed on them. Additionally, the 10 patients in group C were measured in terms of WISP1 protein expression with ELISA.

### Determining the PQ level in the serum

The PQ concentration in the serum of the patients was examined through high-performance liquid chromatography [14]. All 37 patients were examined in terms of serum PQ concentration on day 1 of PQ poisoning. The group C participants were examined in terms of serum PQ concentration as healthy controls.

### The treatment and follow-up of cases

All patients were immediately given gastric lavage upon presenting to the hospital. Subsequently, they received catharsis therapy using activated carbon (20 g) and 20% (w/v) mannitol (250 ml) every 4–6 h. All patients were treated with ulinastatin (100,000 U, intravenous bolus, every 4–6 h) and methylprednisolone (500 mg, intravenous drip, once a day) for 3 days. Some received hemoperfusion therapy. Any changes in the patients’ blood gas analysis index and chest computed tomography (CT) or x-ray images were observed. All of the patients who survived received a follow-up chest CT scan, and their clinical symptoms were reviewed 2 months after they had been poisoned; ALI [15] or PF [16] was determined according to guidelines.

### Statistical analysis

Data were analysed using the SPSS Statistics 19.0 (IBM SPSS, Armonk, NY, USA) software and were presented as the mean ± standard deviation. A two-tailed Student’s t-test was used to analyse the differences between the two groups. The relationships between the categorical variables and the outcomes were evaluated using Spearman’s correlation, a chi-square test and logistic regression; *P* < 0.05 was significantly different.

## Results

### Subject characteristics

Thirty-seven patients (20 females and 17 males) were recruited for this study. The age and intake amount of PQ of the PQ-poisoned patients were 30.97 ± 11.54 years and 59.34 ± 150.90 ml, respectively. Thirteen cases in the PF group had ALI or PF. In the PF group, 9 patients died of acute respiratory failure resulting from poisoning between days 2–7, and 4 patients survived but with PF. All the participants were tested on day 1 of the poisoning. Only 11 patients were tested for the WISP1 gene and protein expression on day 3. The patients in the PF group were older (t = 2.409, *P* = 0.021) and had a higher serum PQ concentration (t = 4.305, *P* = 0.000) than those in the NPF group (Table 2).

**Table 2:** The characteristics of the PF and NPF groups

Group	Sex		Survival		Age (years)	Oral amounts (ml)	PQ concentration (mg/L)
					$\left(\overline{x}\pm s\right)$	$\left(\overline{x}\pm s\right)$	$\left(\overline{x}\pm s\right)$
	Males	Females	Day 1	Day 3			
PF (*n* = 13)	5	8	13	11	36.23 ± 11.77	123.08 ± 245.26	3.81 ± 1.77
NPF (*n* = 24)	12	12	24	24	27.29 ± 10.22	24.50 ± 26.32	2.07 ± 0.68
C (*n* = 10)	5	5			30.25 ± 1.75	0	0
t					2.409*	1.972	4.305**
*P*					0.021	0.057	0.000

*PF* pulmonary fibrosis, *NPF* non-PF, *C group* negative control group. **P < 0.01, compared with patients in the NPF group; *P < 0.05, compared with patients in the NPF group

The PF group also sustained additional organ injuries, compared with the NPF group. The injured organs in the NPF group were the kidneys, indicating acute renal injury. This reached a peak approximately 1 week after poisoning. In addition to kidney injury, the patients in the PF group were prone to liver, pancreas and myocardium injuries.

### Changes in chest x-ray and CT scan images

The primary x-ray changes in the PF group involved alveolar exudation; the x-rays of some patients also showed bronchopneumatic signs. Following treatment, the alveolar cavity exudation decreased, and interstitial PF gradually appeared, reflecting primarily sub-pleural honeycomb-like changes. Changes in the CT images showed typical PQ-poisoned lungs.

### The WISP1 gene expression and serum protein concentration in the two groups

**Fig. 1:**
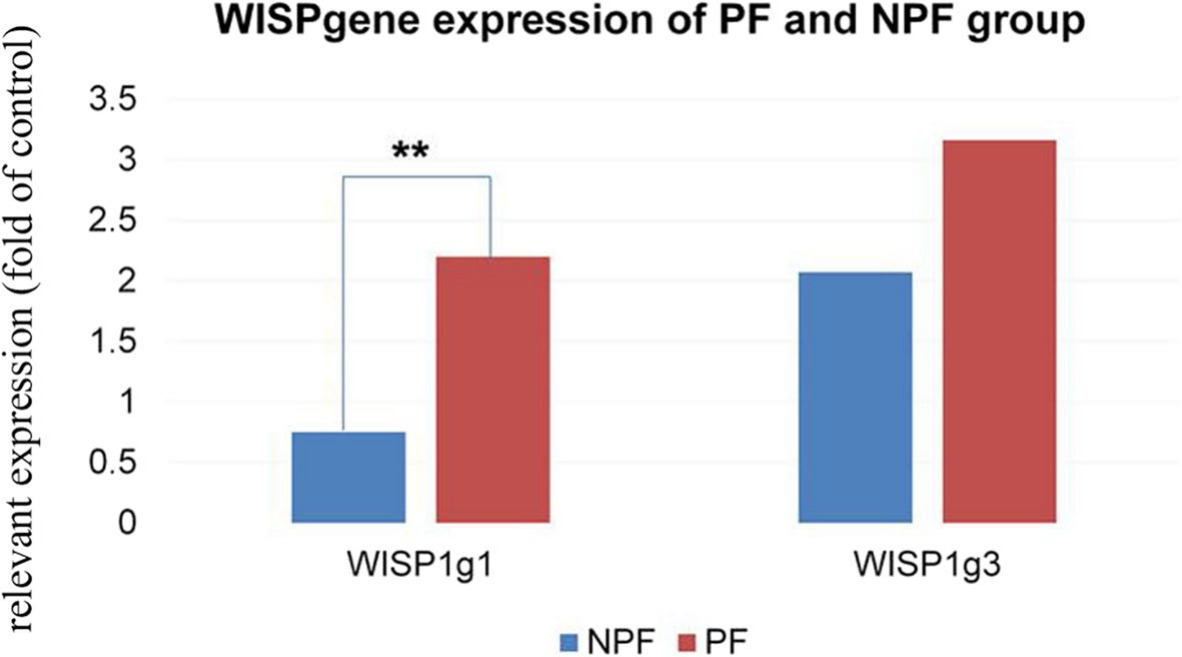
WISP1 gene expression in the PF and NPF groups. PF, pulmonary fibrosis; NPF, non-PF; WISP1g1, WISP1 gene expression on the first poisoning day; WISP1g3, WISP1 gene expression on the third poisoning day. **P < 0.01, compared with patients in the NPF group

The WISP1 gene expression in the PF group was higher than the NPF group on day 1 of poisoning (t = 7.541, *P* < 0.01); in addition, no difference was observed on day 3 (t = 1.243, *P* > 0.05). In the NPF group, it was higher on day 1 than on day 3 (t = 2.335, *P* = 0.028). There was no difference in the PF group between days 1 and 3. The WISP1 gene expression in the PF and NPF groups were all higher compared with group C (Table 3, Fig. 1).

**Table 3:** The WISP1 gene expression for groups C, PF and NPF

Group	Time	Number	Expression of the WISP1 gene $\left(\overline{x}\pm s\right)$	***t***	***p***
NPF	1d	24	0.75 ± 0.12^##^	5.089	0.000
	3d	24	2.07 ± 2.80^*##^	2.335	0.028
PF	1d	13	2.20 ± 0.92^**##^	7.541	0.000
	3d	11	3.16 ± 1.60^##^		
C		10	0.26 ± 0.06		

*PF* pulmonary fibrosis, *NPF* non-PF, *C group* negative control group. ^**^P < 0.01, compared with patients in the NPF group; ^*^P < 0.05, day 3, compared with day 1. ^##^*P* < 0.01, compared with group C (NPF, day 1, t = 5.089, *P* = 0.000, NPF, day 3, t = 3.331, *P* = 0.003; PF, day 1, t = 7.269, *P* = 0.000, PF, day 3, t = 5.656, *P* = 0.000)

**Fig. 2:**
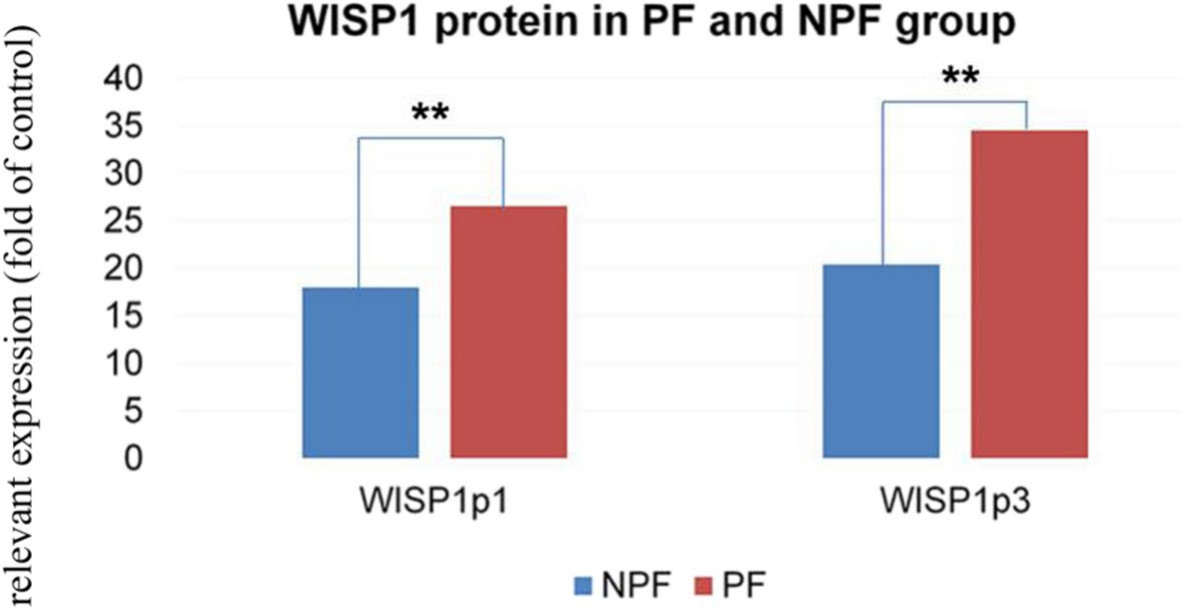
Serum WISP1 protein concentration in the PF and NPF groups. PF, pulmonary fibrosis; NPF, non-PF; WISP1p1, WISP1 protein concentration on the first poisoning day; WISP1p3, WISP1 protein concentration on the third poisoning day. **P < 0.01, compared with patients in the NPF group

The WISP1 protein concentration in the PF group was higher than in the NPF group on both days (t = 7.166, *P* < 0.01 on day 1; t = 5.855, *P* < 0.01 on day 3). On day 3, the WISP1 protein concentration in the PF group was higher than on day 1. There was no difference in the PF group between days 1 and 3. The WISP1 protein concentration of the NPF and PF groups were higher than those in group C on both days 1 and 3 (Table 4, Fig. 2).

**Table 4:** The WISP1 protein of groups C, PF and NPF

Group	Time	Number	Expression of WISP1 protein (pg/ml) $\left(\overline{x}\pm s\right)$	***t***	***p***
NPF	1d	24	17.97 ± 1.04^##^		
	3d	24	20.38 ± 1.01^##^		
PF	1d	13	26.54 ± 5.63^**##^	7.166	0.000
	3d	11	34.51 ± 11.65^****##^	5.855	0.000
C		10	8.07 ± 1.26		

*PF* pulmonary fibrosis, *NPF* non-PF, *C group* negative control group. ^**^P < 0.01, compared with patients in the NPF group; ^**^P < 0.01, compared with day 1, t = 3.253, *P* = 0.002.^##^*P* < 0.01, compared with group C (NPF, day 1, t = 12.924, *P* = 0.000, NPF, day 3, t = 12.919, *P* = 0.000; PF, day 1, t = 10.996, *P* = 0.000; PF, day 3, t = 7.137, *P* = 0.000)

### Factors correlated with PF

A correlation analysis showed that both WISP1 gene expression and serum WISP1 protein concentration were positively correlated with the serum PQ concentration on day 1 of poisoning (*r* = 0.621, *r* = 0.596, all *P* < 0.01). PF was correlated with WISP1 gene expression on day 1 of poisoning (*r* = 0.424, *P* < 0.01), oral PQ amount (*r* = 0.416, *P* < 0.05) and serum PQ concentration (*r* = 0.467, *P* < 0.01). The logistic regression analysis included factors such as patient age, oral PQ amount, serum PQ concentration, WISP1 gene expression and WISP1 protein concentration in the serum of patients in the PF group. The risk factor for PQ-induced PF was serum PQ concentration [OR = 4.356, 95% CI (1.188–15.971), *P* < 0.05]. The results of the adjusted and non-adjusted OR values for WISP1 gene expression and WISP1 protein concentration on day 1 were OR = 12.797, 95% CI (2.478–66.076), *P* = 0.002, OR’ = 11.353, *P* = 0.005 and OR = 1.545, 95% CI (1.197–1.995), *P* = 0.001, OR’ = 1.487, *P* = 0.003.

## Discussion

PQ was widely used in Asian agriculture as an organic heterocyclic herbicide. However, in recent years, reports of its highly toxic nature have emerged. The poisoning rate related to PQ, either accidental or suicidal, has recently shown a significant upward trend [17]. At present, clinical evidence indicates PF is one of the main causes of death following PQ intake [18, 19]. In the currently available research, however, the exact pathogenesis of PF remains unknown. The most effective clinical intervention of PF is immunosuppressive therapy. However, due to a lack of supporting evidence, this intervention has not been widely applied [20, 21]. Therefore, a relevant molecular mechanism must be established that can help clarify the future of clinical intervention for PF.

In this study, 9 patients died of acute respiratory failure, and 4 patients with PF survived. The patients who developed PF had ingested higher oral amounts of PQ and had higher serum PQ concentrations than those that did not develop PF. The serum PQ concentration was a risk factor of PQ poisoning with PF complications. This result was consistent with the observations of Chinese children poisoned with PQ [22]. A logistic regression analysis showed that the oral PQ amount was not a risk factor; however, this may have been the result of the inaccurate estimation of oral PQ amounts because the PQ absorbance differed between the participants. The treatment was also affected by the level of PQ absorbance.

WISP1 is a newly discovered signalling molecule of the CCN family [23] and is up-regulated in humans with idiopathic PF [8, 24]. The concentrations of many cytokines (i.e., TNF-α, IL-6 [25–27] and TGF-β [28]) increased in PQ-poisoned animal models. Additionally, WISP1 is a common downstream target of TGF-β1 and TNF-α in primary human lung fibroblasts; WISP1 also exerts profibrotic functions through IL-6-dependent induction of fibroblast proliferation [29]. Based on these existing studies, it was speculated that WISP1 plays a role in PQ-induced PF through cytokine interactions. This study’s results revealed that the mRNA and protein levels of WISP1 were highly increased in patients with PQ-induced PF.

The results of the study also indicated that patients in the NPF group had higher gene expression on day 3 following poisoning. The possible reasons for this are as follows. When PQ entered the human gastrointestinal tract, gastric lavage could not remove all the poison. Accordingly, a degree of poison was absorbed into the bloodstream and its concentration increased without blood perfusion treatment. PQ is widely distributed in the human body and its half clearance time is 84 h [30]. The concentration of PQ in the lungs and skeletal muscles will be the highest. Because PQ is positively correlated with WISP1 gene expression, the increase of gene expression in the first 3 days following poisoning may also be related to the increase in PQ content in the skeletal muscles. However, this requires further research.

To the knowledge of the authors, this study was the first to determine changes in WISP1 in PQ-poisoned patients. The serum PQ concentration was positively correlated with the WISP1 gene expression and serum WISP1 protein concentration. It was observed that the WISP1 gene expression and protein concentration in PQ-poisoned individuals were higher than in healthy controls. The WISP1 gene expression and WISP1 protein concentration on day 1 of poisoning was a risk factor of PQ-induced PF. The results of this study revealed that PQ could induce WISP1 gene expression and further increase serum WISP1 concentration. Accordingly, WISP1 gene expression may play a role in PQ-induced PF.

### Study limitations

This study was limited in terms of the small number of PQ-poisoned patients who were included. Larger samples and additional treatments should be reviewed in future studies.

## Conclusion

WISP1 was up-regulated in patients with PQ-induced PF, and serum PQ concentration was closely correlated with WISP1 gene expression. Serum PQ concentration was a risk factor of PQ poisoning combined with PF. Therefore, the WISP1 gene may play an important role in PQ-induced PF.

## Data Availability

The datasets used or analysed during the current study available from the corresponding author on reasonable request.

## Abbreviations

WISP1: Wnt-induced secreted proteins-1
PQ: Paraquat
C: Control
ALI: Acute lung injury
CT: Computed tomography
PF: Pulmonary fibrosis
EMT: Epithelial–mesenchymal transition
TGF-ß: Transforming growth factor-ß
TNF-α: Tumour necrosis factor-α
IL-6: Interleukin- 6
CCN: Cyr61, CTGF, Nov
ECM: Extracellular matrix
IPF: Idiopathic pulmonary fibrosis
RT-PCR: Reverse transcription-polymerase chain reaction
NPF: Non-pulmonary complications
ELISA: Enzyme-linked immunosorbent assay
